# HeartEnsembleNet: An Innovative Hybrid Ensemble Learning Approach for Cardiovascular Risk Prediction

**DOI:** 10.3390/healthcare13050507

**Published:** 2025-02-26

**Authors:** Syed Ali Jafar Zaidi, Attia Ghafoor, Jun Kim, Zeeshan Abbas, Seung Won Lee

**Affiliations:** 1 Institute of Information Technology, Khwaja Fareed University of Engineering and Information Technology, Rahim Yar Khan 64200, Pakistan; 2 Department of Metabiohealth, Sungkyunkwan University, Suwon 16419, Republic of Korea; 3 Department of Precision Medicine, Sungkyunkwan University School of Medicine, Suwon 16419, Republic of Korea; 4 Department of Artificial Intelligence, Sungkyunkwan University, Suwon 16419, Republic of Korea; 5 Personalized Cancer Immunotherapy Research Center, Sungkyunkwan University School of Medicine, Suwon 16419, Republic of Korea

**Keywords:** artificial intelligence, cardiovascular prediction, machine learning, ensemble learning, healthcare

## Abstract

Background: Cardiovascular disease (CVD) is a prominent determinant of mortality, accounting for 17 million lives lost across the globe each year. This underscores its severity as a critical health issue. Extensive research has been undertaken to refine the forecasting of CVD in patients using various supervised, unsupervised, and deep learning approaches. Methods: This study presents HeartEnsembleNet, a novel hybrid ensemble learning model that integrates multiple machine learning (ML) classifiers for CVD risk assessment. The model is evaluated against six classical ML classifiers, including support vector machine (SVM), gradient boosting (GB), decision tree (DT), logistic regression (LR), k-nearest neighbor (KNN), and random forest (RF). Additionally, we compare HeartEnsembleNet with Hybrid Random Forest Linear Models (HRFLM) and ensemble techniques including stacking and voting. Results: Employing a dataset of 70,000 cardiac patients with 12 clinical attributes, our proposed model achieves a notable accuracy of 92.95% and a precision of 93.08%. Conclusions: These results highlight the effectiveness of hybrid ensemble learning in enhancing CVD risk prediction, offering a promising framework for clinical decision support.

## 1. Introduction

Cardiovascular disease (CVD) is a predominant driver of fatalities across the globe, responsible for a staggering 17.3 million deaths Year after year [1] as endorsed by the World Health Organization (WHO) and World Heart Federation (WHF) [2,3].

Epidemiological data of health statistics highlight the global burden of CVD, with a particularly high prevalence in low and middle income countries including Russia, India, and China, which share similar demographic patterns. The development of CVD is influenced by multiple risk factors such as obesity, high blood pressure, diabetes, and high cholesterol, compounded by inadequate lifestyle management. Early detection of CVD is substantial to mitigate complications, reduce hospitalizations, and enhance patient outcomes [4,5]. However, the heterogeneity of CVD symptoms across demographics complicates timely diagnosis. For instance, women often exhibit atypical symptoms such as nausea and stomach discomfort, whereas men more commonly report chest pain [6,7,8,9]. These variations necessitate advanced predictive models that integrate multiple risk factors for accurate and personalized diagnosis.

Numerous state-of-the-art (SOTA) academic pursuits aim to enhance the prognostic reliability of CVD detection, comprising deep learning and machine learning frameworks, outperform classical approaches by enhancing cardiovascular disease forecasting, significantly reducing healthcare cost [10,11,12,13]. Unlike conventional statistical models, ML-based classifiers leverage complex patterns in large datasets, improving diagnostic accuracy and reducing healthcare costs [14,15]. This study proposes HeartEnsembleNet, a hybrid ensemble learning framework that integrates multiple classifiers to enhance predictive performance. By comparing it against traditional ML models and state-of-the-art ensemble techniques, we demonstrate its efficacy in cardiovascular risk assessment.

### Problem Statement

This study aims to identify risk factors contributing to cardiac dysfunction in CVD. Despite significant advancements, gaps persist, necessitating a more robust predictive framework. This study employs a hybrid framework that integrates machine learning techniques, encompassing HRFLM and ensemble models (stacking and voting), to address gaps in current CVD prediction methods. The fundamental objective of implementing integrated machine learning models is to empower systems to autonomously identify trends and predict events without human intervention. This study provides novel contributions to cardiovascular risk prediction by:

1. **Designing a Breakthrough Ensemble-Based Prediction Model:**
  This study conducts a comprehensive comparative assessment of cutting-edge ensemble approaches, including voting and stacking, to evaluate the strengths and limitations of Hybrid Random Forest Linear Models (HRFLM) and foundational classifiers. Additionally, a hybrid approach integrating K-Nearest Neighbors (KNN) and Random Forest (RF) techniques is proposed to streamline prediction results.
2. **Proposing a Novel Symptom-Driven CVD Patient Recognition Model:**
  A symptom-centric diagnostic model will be developed to facilitate prompt detection and intervention. The system’s modular design ensures simplicity and applicability, enabling seamless integration with diverse datasets and healthcare settings.

## 2. Materials and Methods

### 2.1. Data Acquisition

This study utilized a high-quality dataset sourced from Kaggle [16], an open-source platform known for its well-curated datasets and data science competitions. Specifically, the dataset was part of a Kaggle competition, which underscores its relevance and utility for research in this domain. Kaggle serves as a valuable resource for exploring datasets and challenges, fostering opportunities for learning and innovation. The dataset comprises clinical records of 70,000 diverse patients diagnosed with cardiovascular disorders, incorporating 12 key parameters reflecting individual patient profiles. These parameters capture essential clinical attributes associated with CVD, enabling a comprehensive investigation of disease risk factors. Each parameter represents a distinct, non-ranged value collected from patients across various age groups. Table 1 presents the selected features considered for experimentation in the proposed HeartEnsembleNet model. Additionally, the dataset lacks critical cardiovascular diagnostic markers, encompassing electrocardiography (ECG), echocardiography, and biomarkers consist of Troponins and NT-ProBNP, which could enhance specificity and clinical relevance if incorporated.

### 2.2. Dataset Preprocessing and Exploratory Data Analysis

**Figure 1:**
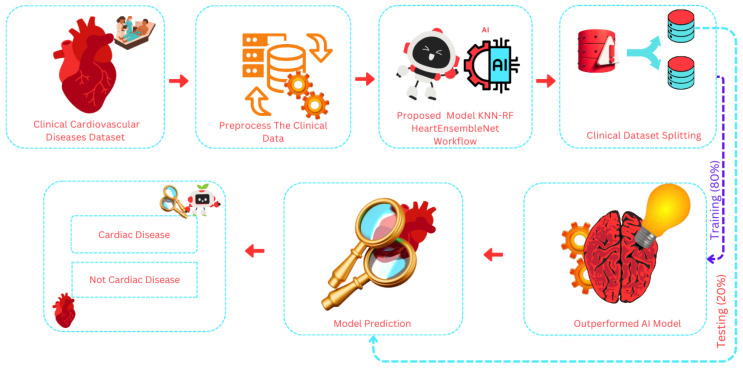
Holistic structural workflow of the innovative approach, HeartEnsembleNet, for cardiovascular disease detection.

**Figure 2:**
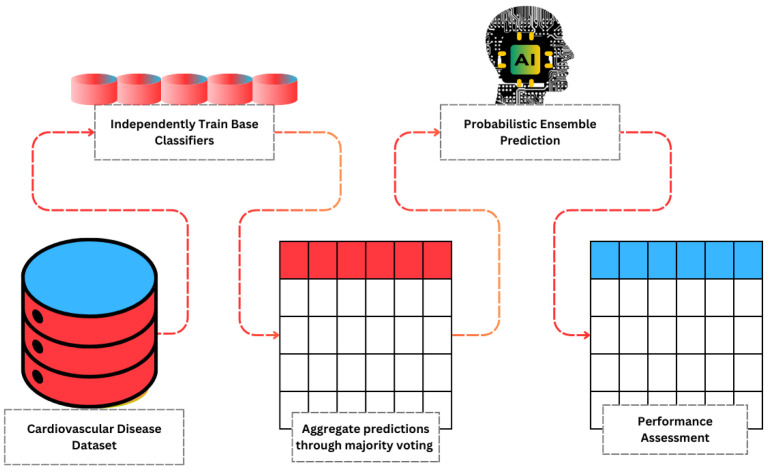
Architectural analysis of novel feature selection approach presented for cardiac failure diagnosis.

To prepare the CVD dataset for machine learning model training, a streamlined data refinement process was applied. Initially, unstructured data was cleaned and transformed into a standardized format to facilitate hypothesis testing. The complete workflow is shown in Figure 1, while Figure 2 showcases a brief architecture analysis of the proposed feature extraction approach.

#### 2.2.1. Skewness and Kurtosis Assessment

**Figure 3:**
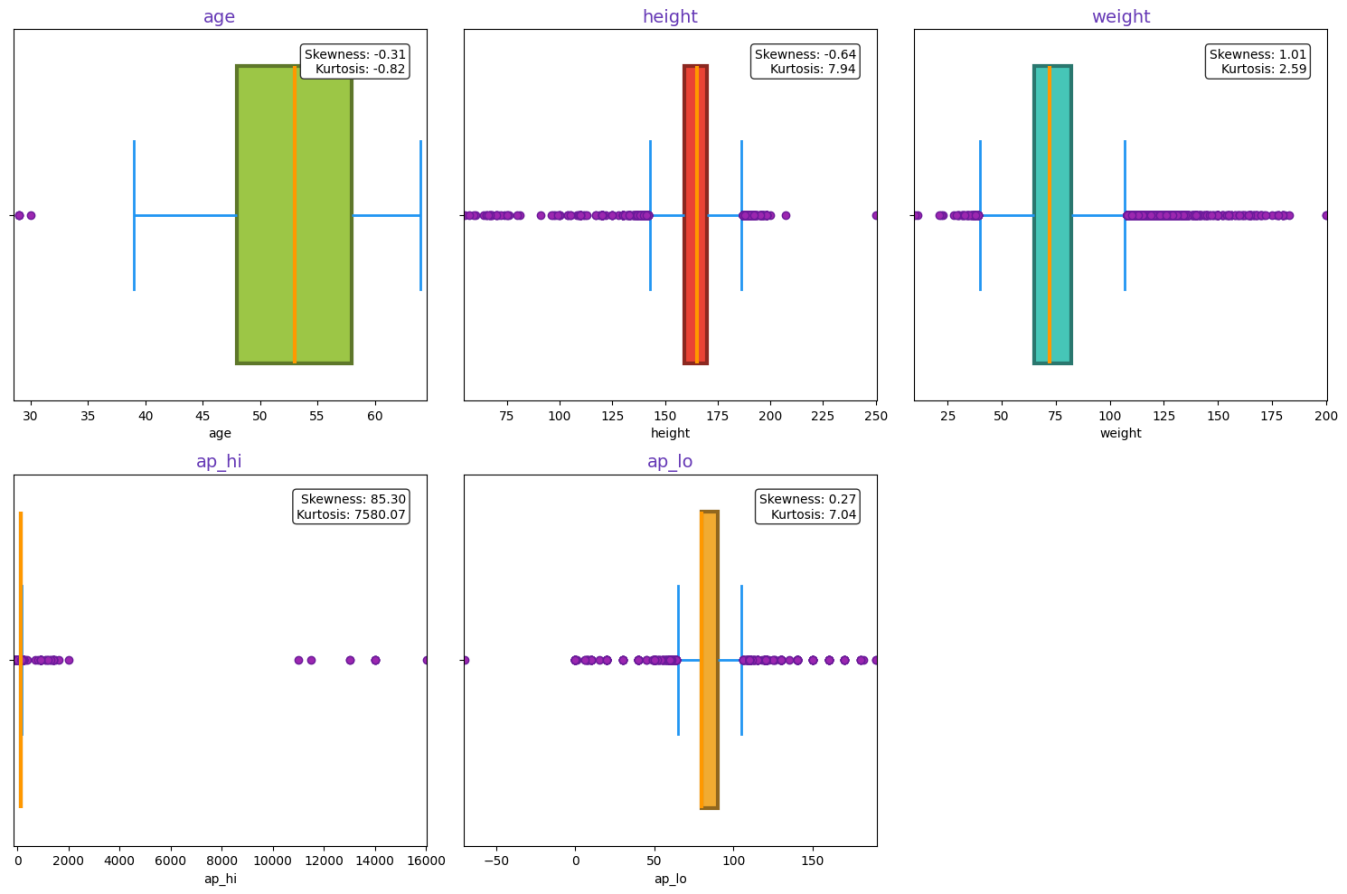
Skewness and Kurtosis evaluation for normality assessment for cardiovascular disease dataset features.

Skewness and kurtosis were assessed to analyze the symmetry and tail behavior of the data distribution in Figure 3. Features with significant skewness were transformed using logarithmic scaling to achieve normality, improving model performance by aligning the data with underlying assumptions of many machine learning models.

#### 2.2.2. Outlier Handling Using IQR Method

**Figure 4:**
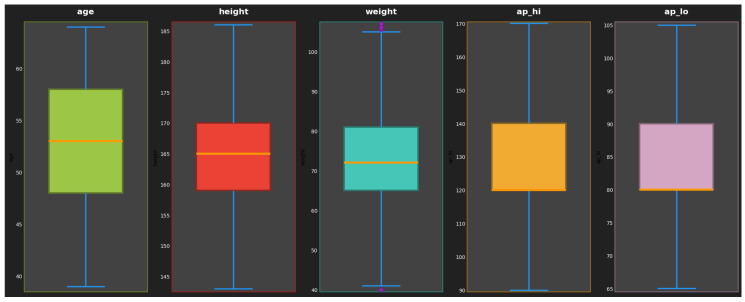
Outlier handling for data quality and model performance in cardiovascular disease dataset.

Outliers were detected and removed using the Interquartile Range (IQR) method, which helps maintain data quality and ensure accurate model training. Data points falling outside the bounds Q1−1.5×IQR and Q3+1.5×IQR were identified as outliers and eliminated to prevent distortion of the dataset in Figure 4.

#### 2.2.3. Feature Scaling and Transformation

Continuous features, such as age, height, and weight, were standardized using the StandardScaler to normalize the data. In some cases, normalization was applied using the MinMaxScaler to scale features within a specific range, ensuring consistency across features with different units.

#### 2.2.4. Data Normalization and Standardization

In cases where normalization was required, the MinMaxScaler was optionally applied to scale values within a specific range. This ensures comparability of features with differing units and magnitudes, further enhancing model stability. Standardization was applied to ensure that each feature has a mean of 0 and a standard deviation of 1, which is particularly important for models like LR or SVM, which assume that the data is normally distributed.

#### 2.2.5. Categorical Variables Encoding

**Figure 5:**
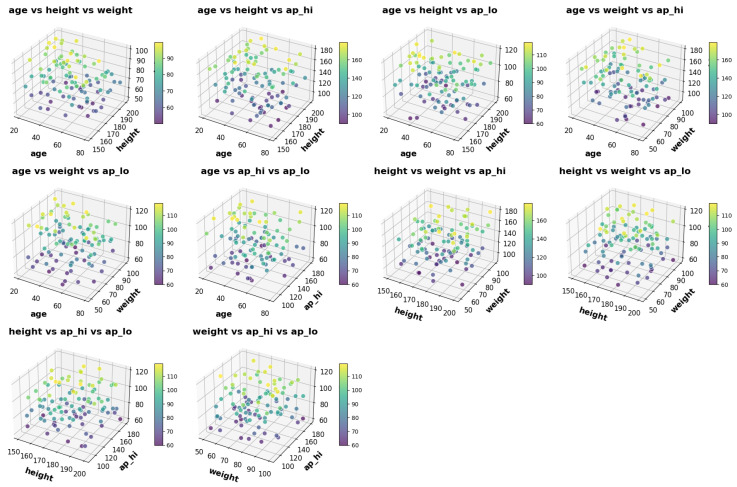
Pair plot of continuous features in the cardiovascular disease datase.

**Figure 6:**
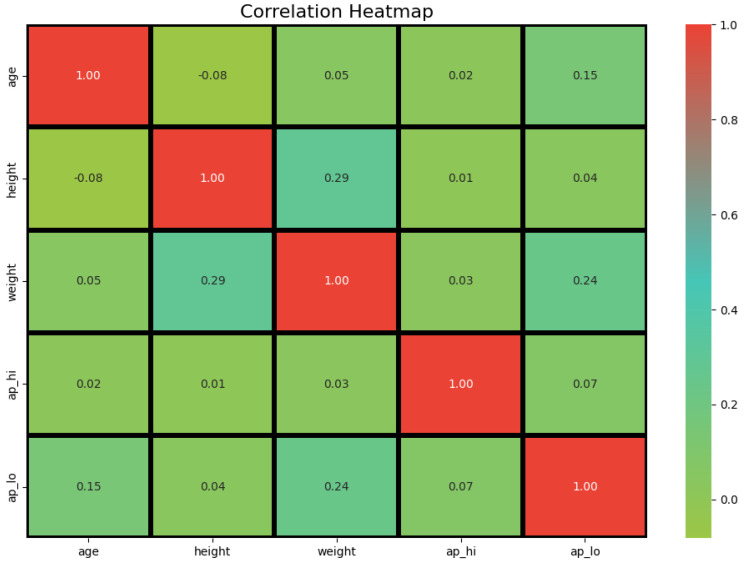
Correlation heatmap of numerical features in the cardiovascular disease dataset.

Categorical features, incorporating gender, cholesterol, gluc, and others, were converted into numerical representations using one-hot encoding. This transformation ensures compatibility with machine learning algorithms that require numeric input. Figure 5 and Figure 6.

### 2.3. Class Distribution Analysis

Class imbalance was visualized and addressed using resampling techniques. The distribution of the target variable before and after resampling was analyzed to ensure that the model training process was not biased. The balanced distribution enables the model to learn from the data effectively and ensure fair performance.

### 2.4. Pairwise Correlation Analysis

Pairplots were employed to analyze relationships between continuous features, while heatmaps were utilized to highlight correlations among numerical features. These visualizations provided valuable insights into feature dependencies and redundancies, as demonstrated in Figures.

### 2.5. Dataset Splitting

The dataset used in this study comprises records of 70,000 patients with 12 distinct attributes. It has been splitted into 80% training and 20% testing ratio to ensure robust evaluation. The following sections provide a detailed discussion of the test results, assessed using comprehensive evaluation metrics.

### 2.6. Classical Machine Learning Models

Our study proposes a ML-based framework for survival rate estimation in cardiovascular disease detection. The proposed approach incorporates six state-of-the-art classification algorithms, including Gradient Boosting (GB), Support Vector Machine (SVM), Logistic Regression (LR), k-Nearest Neighbors (KNN), Decision Tree (DT), and Random Forest (RF) [17,18,19]. Classical ML classifiers have been widely applied across various domains to address complex problems such as predictive modeling, clustering, and pattern recognition. These techniques are particularly valued for their computational efficiency, especially when handling moderately sized and well-structured datasets.

#### 2.6.1. Support Vector Machine (SVM)

SVM, formulated in 1995, is a versatile model that utilize kernel methods for both classification and regression tasks [20]. SVM determine optimizing hyperplane configurations in a multidimensional domains to segregate data into distinct categories efficiently. Margin maximization is applied to identify the hyperplane parameters. The margin quantify the shortest gap between hyperplane and the closest point (Support Vectors). The hyperplane’s dimensionality directly correlates and dictates hyperplane geometry.

The prediction function for Support Vector Classifier is:

(1) $$f\left(x\right)=w^{T}x+b$$

Classification involves employing the decision formula

(2) $$\hat{y}=\{\begin{array}{cc} +1 & \mathrm{if}\;f(x)>0\\ -1 & \mathrm{if}\;f(x)<0 \end{array}$$

where;

- f(x) denotes the predicted classification result.
- *w* presents weight vector,
- *x* is the observation vector,
- *b* is the constant parameter,
- $\hat{y}$ is the estimated class label.

#### 2.6.2. Random Forest (RF)

Random Forest classifiers utilize an ensemble of decision trees derived from bootstrap samples of data significantly, boosting robustnes and generalization to novel situations. By aggregating the results of individual tree predictions, the ensemble methods optimize model robustness and predictive performance [21].

(3) $$\hat{y}=\mathrm{majority\_vote}\left(T_{1}\left(x\right),T_{2}\left(x\right),\cdots ,T_{m}\left(x\right)\right)$$

(4) $$\hat{y}=\frac{1}{m}\sum_{i=1}^{m}T_{i}\left(x\right)$$

here;

- Ti(x) is the estimated value from the *i*th decision tree for input feature vector *x*,
- majority_vote utilized for classification and the ensemble’s output is identified by the majority vote
  $$\frac{1}{m}\sum \left[T_{i}\left(x\right)\right]\;\mathrm{applied}\;\mathrm{for}\;\mathrm{predictive}\;\mathrm{mean}\;\mathrm{calculation}$$

#### 2.6.3. Decision Tree (DT)

DT, a supervised machine learning technique employs a hierarchical segmentation approach addressing regression and classification issues in multifaceted domains [22]. DT employ a top down approach to facilitate intricate decision-making process depicted in a dendrogrammatic comprising nodes and edges representation for optimal attribute selection.

**For classification:**

1. Evaluate feature values of *x* by recursively traversing the tree nodes.
2. The leaf node prediction is determined by majority voting among the instances within that node.

This concept can be expressed as:

(5) $$\hat{y}=\mathrm{majority\_class}\left(\mathrm{leaf}\;\mathrm{node}\;\mathrm{containing}\;x\right)$$

where:

In the classification context, $\hat{y}$ is the predicted class label, determined by the majority class in the leaf node containing the input *x*.

**For regression:**

1. Recursively traverse the tree until reaching a terminal leaf node.
2. The prediction is the average of the target values in that leaf node.

This sentiment can be framed as:

(6) $$\hat{y}=\frac{1}{N}\sum_{i=1}^{N}y_{i}$$

In the regression context, $\hat{y}$ constitutes the predicted value, derived from the arithmetic mean of target variables encompassing input *x*. Here, *N* showcases the sample count in the leaf node paired with target values denoted by $y_{i}$.

#### 2.6.4. K-Nearest Neighbor (KNN)

KNN is a versatile and widely employed non-parametric approach utilized in numerous classification and regression analysis [23]. It operates based on the concept of spatial proximity-based categorization to classify data points rooted in the framework of dominant class of neighboring instances. Three fundamental steps underlies the algorithm’s framework incorporating Euclidean, Minkowski or Manhattan metrics significantly influences model performance.

The conceptual framework of Euclidean distance metric is represented as

**Euclidean Distance**

$$d\left(x,y\right)=\sqrt{\sum_{i=1}^{n}{\left(x_{i}-y_{i}\right)}^{2}}$$

here;

- *n* signifies the dimensionality.
- x,y represents coordinate pairs of data points, and $x_{i}$ and $y_{i}$ denote the *i*th dimensions.
- ${\left(x_{i}-y_{i}\right)}^{2}$ quantifies the squared discrepancy across analogous dimensions.

**Manhattan Distance**

$$d\left(x,y\right)=\sum_{i=1}^{n}\left|x_{i}-y_{i}\right|$$

- The term $|x_{i}-y_{i}|$ serves as a measure of absolute feature disparity.

**Minkowski Distance**

$$d\left(x,y\right)={\left(\sum_{i=1}^{n}{\left|x_{i}-y_{i}\right|}^{p}\right)}^{\frac{1}{p}}$$

here;

- *p* is a hyperparameter significantly impacts distance-based analysis.
- The Minkowski distance p=1 precipitates a transition to Manhattan distance.
- When p=2 the Minkowski distance facilitates the conversion to Euclidean distance.

**Neighbor Selection**

$$\mathrm{Neighbors}={\mathrm{argmin}}_{x_{i}\in \mathrm{Training}\mathrm{Set}}\;\left\{d\left(x,x_{i}\right)\right\}$$

- This approach determining the *k* most closest data points with user adjustable input parameters critically impacting the model’s robustness.

**Classification**

$$\hat{y}=\mathrm{mode}\left(\left\{y_{1},y_{2},\ldots ,y_{k}\right\}\right)$$

- Classification is performed incorporate majority voting scheme identify the class dominance among adjacent instances.

**Regression**

The formula shows the KNN regression estimator formula formalized as:

$$\hat{y}=\frac{1}{k}\sum_{i=1}^{k}y_{i}$$

#### 2.6.5. Gradient Boosting Classifier (GB)

The GB Classifier showcases considerable noteworthy efficacy is a supervised ML model applied for predictive classification. It employs a gradient boosting approach Integrating multiple base classifiers yielding superior and accurate predictive model [24].

Mathematically GB can be formulated as:

(7) $$\hat{y}=F_{M}\left(x\right)=\sum_{m=1}^{M}\gamma_{m}h_{m}\left(x\right)$$

here;

- *x*: Input feature vector; while, $\hat{y}$: Predicted output of the model.
- $F_{M}\left(x\right)$: Overall model prediction function.
- $\gamma_{m}$: Weight of the *m*-th base model.
- $h_{m}\left(x\right)$: Output of the *m*-th base model.
- $\sum_{m=1}^{M}$: Summation over all base models.

#### 2.6.6. Logistic Regression (LR)

LR is a broadly applied statistical and machine learning technique for categorization tasks, that yields probabilistic predictions for multiple classes [25]. LR is the logistic function, which represents the probability of the positive class:

(8) $$p\left(y=1|x\right)=\sigma \left(z\right)=\frac{1}{1+e^{-z}}\;\mathrm{where}\;z=\beta_{0}+\beta_{1}x_{1}+\beta_{2}x_{2}+\ldots +\beta_{n}x_{n}$$

- p(y=1∣x): The probability of the positive classification outcome.
- $\beta_{0}$: Constant term (intercept).
- $\beta_{1},\beta_{2},\ldots ,\beta_{n}$: Regression coefficients that capture feature effects for $x_{1},x_{2},\ldots ,x_{n}$.

Sigmoid function applied to transform *z* into probability values utilizing the logistic function.

### 2.7. Evaluation Parameters

Classification tasks necessitate the utilization of standardized evaluation parameters for assessing classification model efficacy. To assess the performance of the applied approach this study employed fundamental performance metrics including accuracy, precision, recall and F1 score. The subsequent equations are calculated utilizing the following performance metrics. Where; TP, TN, FP, and FN are True Positive, True Negatives, False Positives, and False Negatives, respectively.

#### 2.7.1. Accuracy

This metric evaluates performance metric that quantifies the proportion of correctly classified instances offering valuable insights of model’s reliability and trustworthiness. Formally represented as:

$$\mathrm{Accuracy}=\frac{\mathrm{TP}+\mathrm{TN}}{\mathrm{TP}+\mathrm{TN}+\mathrm{FP}+\mathrm{FN}}$$

#### 2.7.2. Precision

Precision is an assessment parameter that measures the ratio of true positive predictions across all positive predicted instances. Mathematically, precision is described below:

$$\mathrm{Precision}=\frac{\mathrm{TP}}{\mathrm{TP}+\mathrm{FP}}$$

#### 2.7.3. Recall

Recall, or Sensitivity, analyze the model’s capability to precisely identify positive instances. Recall is a fundamental metric that measures the ratio of accurately recognized positive instances. Recall can be defined as:

$$\mathrm{Recall}=\frac{\mathrm{TP}}{\mathrm{TP}+\mathrm{FN}}$$

#### 2.7.4. F1-Score

F1-Score integrates Precision and Recall to yield a comprehensive model performance. This performance metric is well-suited in mitigating the impact of class imbalance. It is computed as the harmonic average of precision and recall. Mathematically, F1 Score metric can be quantified by employing the below equation:

$$F1\;\mathrm{Score}=2\cdot \frac{\mathrm{Precision}\cdot \mathrm{Recall}}{\mathrm{Precision}+\mathrm{Recall}}$$

### 2.8. Presented Study Computational Setup

The computational setup for model development utilizes Python, version 3.7, leveraging libraries such as TensorFlow, Scikit-learn, and Keras for machine learning tasks, while data manipulation is performed using Pandas and NumPy. The models are trained and evaluated on a high-performance system. Table 2 provides a comprehensive summary of the experimental computational setup used in the Google Colab environment to design and implement CVD detection experiments. This subsection outlines the computational hardware and system specifications that supported the analysis and model development.

### 2.9. Hybrid Random Forest Linear Model

This study highlights how the integration of data-mining approach in the context of predictive modeling substantially revolutionized the domain. A prominent HRFLM, integrates the synergies of RF and linear models employing a hybrid framework to achieve superior predictive performance. By utilizing presented hybrid approach, HRFLM optimally utilizes the intrinsic characteristics of machine learning models. To assure high-fidelity forecasts, HRFLM incorporates diverse input parameters, applied fusion of linear and non-linear features. The conceptual foundation of the proposed framework employs RF in parallel with a linear model facilitates augmented prediction, minimizing overfitting, enhance model generalization. Notably, RF is broadly recognized as the intrinsic characteristics of machine learning models [26]. To assure high-fidelity forecasts, HRFLM incorporates diverse input parameters, applied fusion of linear and non-linear features. The conceptual foundation of proposed framework employ RF in parallel with a linear model facilitates augmented prediction, minimizing overfitting, enhance model generalization consequently ensures more reliable decision making [27]. The efficacy of classification approach can vary significantly, highlighting the need for rigorous evaluation to determine the optimal approach for a given domain. By leveraging aggregating predictions from primary and secondary models, HRFLM dramatically increases the reliability and robust classification paradigm for cardiac failure.

### 2.10. Ensemble Techniques

Multiple models are combined, incorporating ensemble techniques to obtain more precise and improved outcomes for the system that has been suggested. Although an ensemble approach may attain more accurate results than a single classifier or technique would [28,29]. The two ensemble strategies employed in this encompassing voting and stacking to boost classification performance has been discussed below.

#### Stacking and Voting

**Level 0** (Base Models in Stacking): This level used six base models, encompassing SVM, RF, DT, KNN, GBM and LR are trained independently on the CVD dataset. Each individual model extracts complex features from data insights enabling efficient predictions. Ensemble predictions are applied for subsequent ensemble layers as input features.

**Level 1** (Meta-Learner in Stacking): In this level, a meta-learner, selecting a meta-model typically entails from shallow to sophisticated models like LR to GB approach, is utilized combining the strengths from the base model results. Optimal prediction fusion exploiting the expertise of the base models refined by meta-model learning generating accurate and fine-tuned results.

**Voting Layer**: Voting is an profoundly effective ensemble learning approach applied to augment predictive performance by consolidating results from heterogeneous models. It employs prediction aggregation from multiple foundational algorithms capitalizing on diverse model insights significantly producing robust and superior predictive performance. Voting can be applied to both classification and regression tasks.

## 3. Results

### 3.1. Results of Classical ML Approach

**Figure 7:**
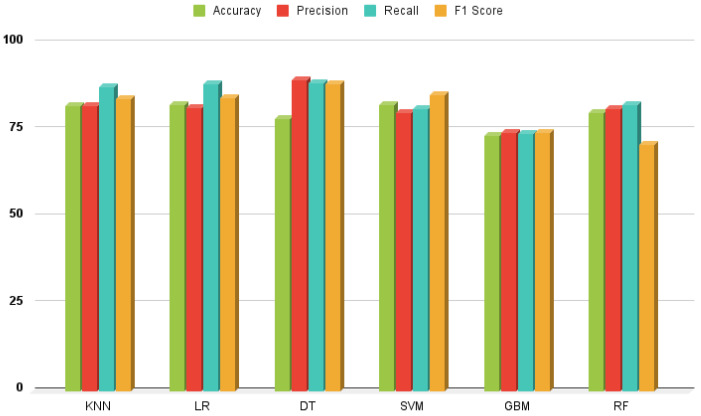
Classical machine learning approach performance analysis.

This section presents a detailed performance analysis of classical machine learning models using accuracy, precision, recall, and F1-score as evaluation metrics. As shown in Figure 7, the models exhibited varying levels of efficacy. A comprehensive assessment was conducted to identify model strengths, limitations, and overall predictive validity. Among all models, SVM, KNN, and LR achieved the highest accuracy scores of 82.33%, 82.10%, and 82.22%, respectively. Notably, SVM demonstrated the best overall performance with an accuracy of 82.33%, precision of 80.11%, recall of 81.11%, and an F1-score of 85.33%. In contrast, GBM recorded the lowest performance, with accuracy, precision, recall, and F1-scores of 73.34%, 74.22%, 74.11%, and 74.34%, respectively. The evaluation results confirm that SVM outperformed other classifiers, achieving the highest recall, accuracy, and F1-score, making it the most effective model in this study.

### 3.2. Results of Ensemble Approaches

**Figure 8:**
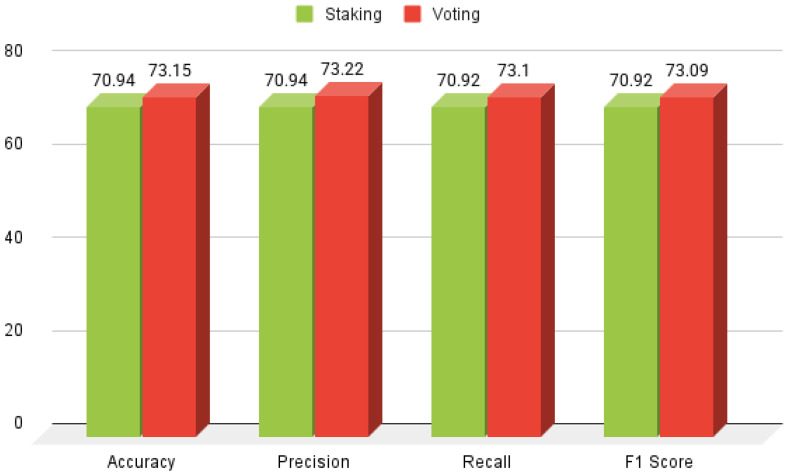
Performance analysis of stacking and voting classifiers.

This section evaluates the performance of voting and stacking ensemble classifiers, which aggregate predictions from multiple models to enhance accuracy. A comprehensive assessment was conducted using precision, accuracy, recall, and F1-score for models incorporating KNN, DT, GB, LR, SVM, and RF. The results, presented in Figure 8, demonstrate the effectiveness of ensemble learning in improving predictive performance.

### 3.3. Results of Hybrid Random Forest Linear Models

**Figure 9:**
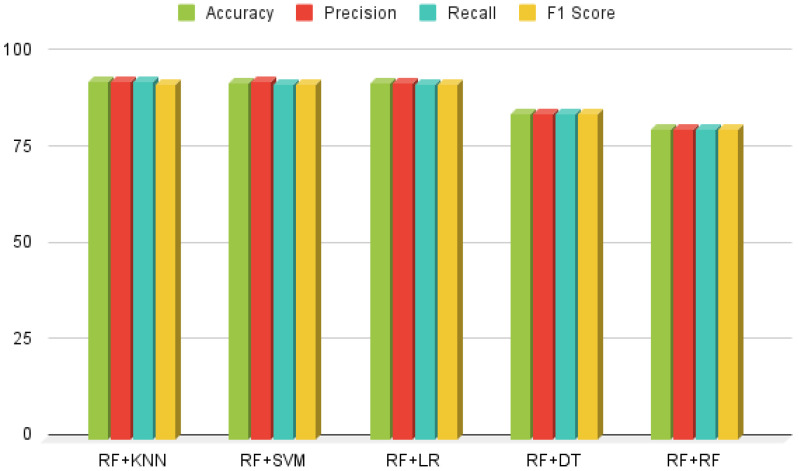
Holistic analysis of Hybrid Random Forest Linear Model.

HRFLM presents a novel fusion of Random Forest and linear models, integrating SVM, DT, RF, KNN, and LR to optimize their synergistic effects. The results presented in Figure 9 the hybrid RF-KNN approach exhibits outstanding classification performance, achieving 92.95% accuracy, 93.08% precision, 92.88% recall, and a 92.47% F1-score. In contrast, the Hybrid RF-DT ensemble demonstrates suboptimal performance with an accuracy of 84.66%. Empirical evidence confirms that HRFLM outperforms conventional machine learning classifiers, delivering significant efficiency improvements. This study underscores the effectiveness of hybrid approaches in heart disease prediction.

### 3.4. Results of Voting and Stacking Approach

**Figure 10:**
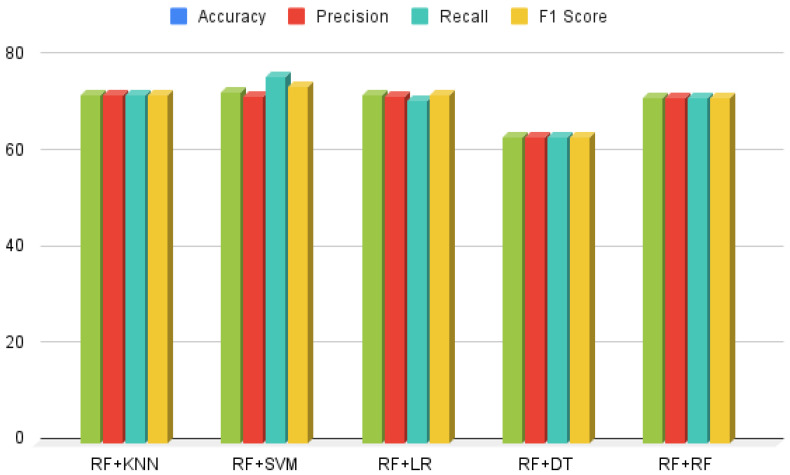
Ensemble voting classifier performance analysis.

**Figure 11:**
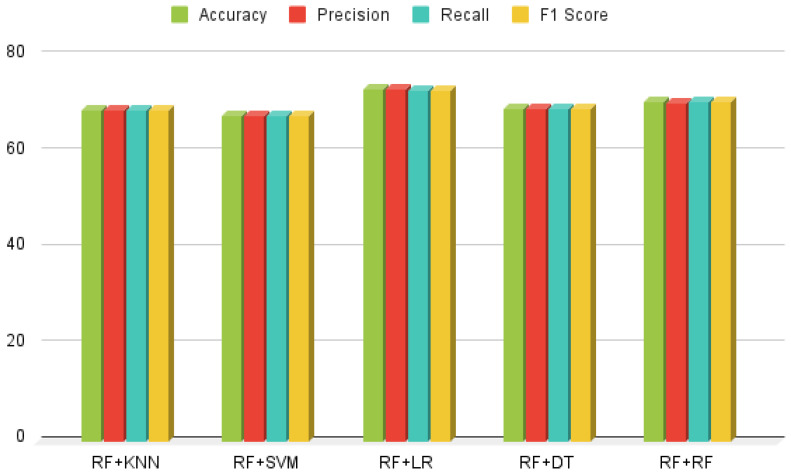
Ensemble stacking classifier performance aanalysis.

Voting and stacking ensemble approaches were employed to comprehensively assess the performance of the Hybrid Random Forest-Logistic Model. Figure 10 and Figure 11 illustrate the implementation of these techniques, which significantly enhanced the performance of HeartEnsembleNet by integrating predictions from multiple models and consolidating their strengths. The combination of Random Forest and Logistic Regression yielded outstanding results using the stacking ensemble, achieving a precision of 73.25%, along with an accuracy of 73.15%, recall of 73.09%, and an F1-score of 73.08%. In contrast, the ensemble voting approach, combining Random Forest and Support Vector Machine, demonstrated the best overall performance, achieving 72.86% accuracy, 72.18% precision, 76.22% recall, and a 74.21% F1-score. A systematic comparative analysis revealed that the voting approach outperformed the stacking method, attaining an accuracy of 73.15%, compared to 70.94% for stacking.

## 4. Discussion

The convergence of healthcare and technology has spurred paradigm-shifting innovations in cardiovascular disease research. Machine learning models have played a crucial role in advancing diagnostic techniques and patient stratification strategies, significantly impacting the early detection of heart diseases, a leading cause of mortality globally. Emerging machine learning frameworks are enhancing the ability to detect nuanced associations and risk factors, improving cardiovascular health assessments. These findings are consistent with our study, which leverages advanced machine learning methods to identify critical predictors of cardiovascular disease. Table 3 shows the result of each classifier used in this study.

### 4.1. Emerging Trends and Breakthroughs in Cardiovascular Disease Research

The fusion of healthcare and technology has fueled paradigm-shifting innovations, igniting groundbreaking and evidence-based practices in state-of-the-art (SOTA) methods. These forward-thinking innovations substantially elevate the refining and optimizing of diagnostics to attain patient stratification breakthroughs. The devastating ramifications of cardiovascular disease pertain to millions of families per year. Prompt identification is a lifesaving strategy; notably, innovative machine learning approaches act as a game-changer in the quest of various diseases, including cancer, brain disorders, kidney issues, and more especially cardiac failure [30] for timely diagnosis. Ground-breaking machine learning frameworks bridge the gap and shine a light on nuanced associations and risk factors that pioneer a new era in cardiovascular health assessment. Landmark research showcases that intelligent models can dramatically elevate especially, utilizing diverse data sources for therapeutic care and lifestyle information, journeying a healthier horizon.

El-Hasnony et al. [31] presents hybrid machine learning and active learning (AL) framework, mitigating the annotated data hurdle through predictive modeling and reduced annotation burden. They conduct comparative analysis of five fold multi-class active learning methods by utilizing grid search hyperparameter sensitivity analysis to amplify model performance. The proposed model encompassing Adaptive, QUIRE, MMC, AUDI and Random for minimizing annotation cost in multi-label classification systematically querying labels targeting most informative data points.

In line with these innovations, our study incorporates similar machine learning models to predict cardiovascular risk. We have found that leveraging diverse datasets, such as clinical records and lifestyle data, significantly enhances model accuracy. This aligns with the work of Yadav et al. [32], who utilized the Heart Disease dataset from the UCI Machine Learning Repository, comprising 14 salient features, to forecast cardiovascular risk. Their study employed seven state-of-the-art machine learning algorithms, including LR, KNN, NB, DT, GB, SVM, and RF classifiers. To evaluate model reliability, the dataset was divided into a 30–70% train-test split. The inter-model comparison demonstrated KNN as the top-performing model, achieving the highest accuracy score of 85.18%, with precision, recall, and F1-score values of 0.83%, 0.90%, and 0.86%, respectively. This study highlights the value of integrating multiple models to boost predictive reliability in future work.

Ref. [33] showcases significant advancements in a refined *k*-modes clustering approach utilizing Huang’s initialization strategy to optimize classification effectiveness. The study employed diverse classifiers encompassing XGBoost, DT, Multilayer Perceptron (MLP), and RF. The machine learning classifiers were fine-tuned on open source dataset from Kaggle consisting 70,000 attributes enabled the MLP technique to hit the mark by yielded 87.28% accuracy score, underscoring the potential of classical machine learning in tackling misdiagnosis substantially, reduce fatalities.

Hossain et al. [34] explores substantial predictive features to uncover intricate patterns in clinical record for cardiac disease risk stratification. A self collected comprehensive dataset was compiled from 59 hospital-admitted patients in Bangladesh incorporating clinical data with qualitative interview data.The study employed a cutting-edge correlation-based feature selection approach along with Best First Search algorithm for enhancing personalized patient care and employed a suite of AI framework namely KNN, LR, NB, DT, random forest ensemble, SVM and MLP neural network on self collected dataset with original and transformed data sets. Their proposed model random forest classifier with extracted features showed better performance, quantify 90% accuracy score with, recall 100% and 90.91% precision and F1-score rendering it a promising solution for cardiac disease detection. Applied conventional machine learning approaches by Ishaq et al. [35] proposed a novel solution for estimating the probability of survival for CVD patients, meanwhile the unbalanced data was addressed using the SMOTE strategy.

The study [36] presents five different risk levels for **cardiovascular disease (CVD)** progression, which have been anticipated in the proposed approach. The five risk levels—No risk, Low risk, Moderate risk, High risk, and Extremely High Risk—have been predicted with 86% accuracy. Chicco et al. [37] outlines a complex algorithm Apache Kafka and Apache that predicts heart disease. Additionally the top contributing features were assessed for the prediction of cardiac disease. Hyperparameter tuning was performed for model assessment and to find the optimal hyperparameters. Also, they applied author employed cross-validation techniques to assess reliability of machine learning classifier with an accuracy score of a 92% on limited data samples.

The study [38] utilized heart disease dataset from IEEE dataport, applied a novel consolidation of five disparate datasets incorporating Cleveland, Long Beach VA, Hungarian, Switzerland and Statlog (Heart) datasets utilizing 1190 patients data. The aggregated dataset offers unparalleled and nuanced understanding. By employing advanced Classification and Regression Tree (CART) approach. Prior to decision tree model training, dataset preprocessing was employed notably, identifying data irregularities as well as removed 272 duplicate data instances, ensuring a robust dataset. The proposed study seeks to forecast heart disease susceptibility and distill transparent decision rules showcasing the interconnections between predictive parameters and prediction breakthroughs. DT performance was assessed by employing evaluation parameters using accuracy, True Positive Rate (TPR), True Negative Rate (TNR) and precision 87%, 85%, 90% and 88% correspondingly.

The investigation Sudha et al. [39] discusses innovative techniques integrating CNN and LSTM networks to surpass the unparalleled accuracy of conventional AI strategies. The dataset used in this research study originates from the UCI machine learning repository, data acquired collaboratively by Clevel and Clinic Foundation and Hungarian Institute of Cardiology. Analyzing a robust dataset 76 key factors spanning 303 records. Data preprocessing performed to elevate model assessment encompasses, missing value imputation utilizing z-score normalization and feature selection were performed using Gini Index, Information Gain, and SVM-weighted methods and PCA for dimensionality reduction techniques. Their proposed model quantify evaluation accuracy score of 89%, sensitivity 81% and specificity 93% versus SOTA classifiers.

A research by Ogundepo et al. [40] employed two complementary open source datasets, namely Cleveland and Statlog. The Cleveland dataset served as the cornerstone of model development and refinment, on other hand, validation was conducted utilizing ten-fold cross-validation on Statlog dataset. Exploratory data analysis, incorporating Chi-square tests, showcase substantial association between High Blood Pressure and Hyperlipidemia, the integration of both elevating cardiovascular disease risk. They implemented ten classification approaches including XGBTree, Conditional random forest, Logit and KNN. SVM outperformed other models, quantify an accuracy score of 85% sensitivity 82%, 87% precision, ROC-AUC: 91% in parallel a minimal log loss 0.38 yielded exceptional results.

**Figure 12:**
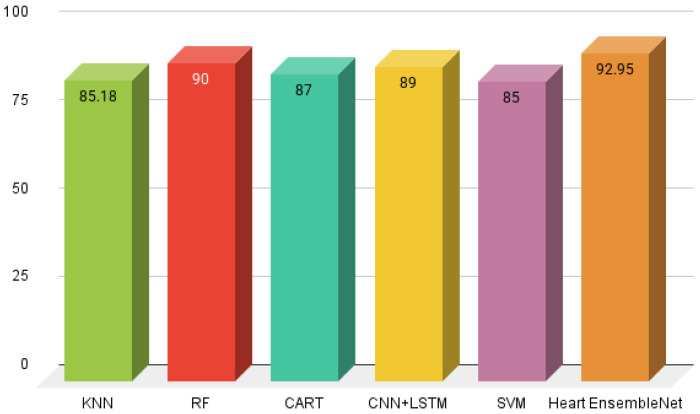
Comparative analysis with SOTA techniques.

The a study, Manikandan et al. [41] incorporates Boruta feature selection approach to optimize feature extraction from the benchmark dataset Cleveland Heart Disease hosted on Kaggle a well known open source plateform. They utilized boruta feature selection algorithm distilled the dataset to six dominant features possessing the greatest influence of the data’s variability captured by the optimal feature subset. This investigation benchmarks the efficacy of six prominent classifiers for cardiac disease, can be seen in Figure 12.

The study Mohapatra et al. [42] outlines a framework for heart disease prediction utilized a two-fold classification strategy that incorporating a diverse range of machine learning models at the core and advanced levels. The developed framework showed a high accuracy score of 92%, coupled with a highest precision of 92.6%, underscoring its exceptional reliability and efficacy. The model’s efficacy was evaluated by employing a suite of metrics incorporating precision, recall, and accuracy. Table 4 summarizes the whole literature, while a novel hybrid deep learning model CNN-LSTM is proposed by [43] to predict cardiac disease utilizing clinical data. This approach leverages features identification, discerns temporal relationships and dependencies across time and interpretable AI SHAP technique for transparency of results of CNN and LSTM. The model attained 74.15% without feature engineering technique.

### 4.2. Research Gap

Brief results of different scholary research has been shown in Table 4. While voting and stacking classifiers have shown promising results, further exploration is needed to develop more robust and accurate CVD prediction models. The study [32] shows that integrating multiple weak models can enhance predictive reliability. However, the integration of advanced architectures, such as transformers, for CVD prediction remains underexplored. In particular, limited efforts have been made to combine transformers with CNN-LSTM models to improve both accuracy and interpretability. Additionally, most studies rely on publicly available datasets, such as UCI, Cleveland, and Statlog, which are often constrained by size, diversity, and outdated representations. These limitations hinder model generalizability across diverse populations and clinical conditions. To address these gaps, our research conducts an in-depth investigation into ensemble machine learning for CVD prediction. Despite dataset constraints, the proposed HeartEnsembleNet model aims to enhance cardiovascular risk stratification through proactive intervention.

## 5. Conclusions and Future Work

Cardiovascular Disease (CVD) remains a major global health concern, necessitating advanced predictive models for early diagnosis and risk assessment. Traditional machine learning techniques have been widely explored for identifying key risk factors; however, their predictive capabilities remain limited due to the complexity of disease patterns. In this study, we proposed HeartEnsembleNet, a novel hybrid ensemble learning framework integrating voting and stacking techniques to enhance predictive performance. Our results demonstrate that HeartEnsembleNet outperforms conventional machine learning classifiers, achieving an accuracy of 92.95%, along with high precision, recall, and F1-score. The integration of stacking methods significantly improves classification performance, demonstrating the potential of hybrid ensemble learning in clinical applications. Furthermore, this approach is adaptable beyond CVD detection, with potential applications in brain stroke prediction, kidney disease identification, and diabetes analysis. For future research, we propose the following directions by expanding biomedical applications by evaluating the adaptability of HeartEnsembleNet in the diagnosis of neurological disorders and cancer, further broadening its scope in medical research. Incorporating deep learning and transfer learning techniques to enhance predictive accuracy, robustness, and generalizability.

### Limitations

Despite its promising performance, HeartEnsembleNet has several limitations that must be addressed for broader clinical applicability. The model is designed for generalized CVD risk assessment rather than distinguishing specific conditions such as coronary artery disease or heart failure, necessitating the development of condition-specific models for improved diagnostic precision. Furthermore, while the model demonstrates strong predictive capabilities, advancements in feature selection and interpretability are necessary for real-world clinical adoption. Integrating interpretable AI techniques will enhance model transparency, fostering greater trust and usability in healthcare settings. it will also contribute to the development of more precise, and clinically relevant cardiovascular risk prediction models, and improving in early detection and personalized treatment strategies.

## Figures and Tables

**Table 1:** Prominent features of Cardiovascular Disease (CVD) dataset.

Sr. #	Attributes	Description
1	Age	Age in years
2	Gender	1: Woman, 2: Man
3	Height	Height in centimeters
4	Weight	Weight in kilograms
5	Systolic Blood Pressure	Systolic BP > 140 mmHg as high
6	Diastolic Blood Pressure	Diastolic BP < 60 mmHg as low
7	Cholesterol	1: Normal (cholesterol ≤ 200 mg/dL), 2: Above normal (200–239 mg/dL), 3: Well above normal (≥240 mg/dL)
8	Glucose	1: Normal (fasting glucose ≤ 100 mg/dL), 2: Above normal (100–125 mg/dL), 3: Well above normal (≥126 mg/dL)
9	Smoking	1: Smoker, 0: Non-smoker
10	Alcohol intake	1: Moderate intake (1–2 drinks/day), 2: Above normal intake (3+ drinks/day), 3: Low/no intake (0 drinks/day)
11	Physical Activity	1: Regular physical activity (≥3 times/week), 0: No regular physical activity
12	Cardiovascular disease	1: Active disease (diagnosed with CVD), 0: No active disease (no CVD diagnosis)

**Table 2:** Computational setup employed in google colab environment for cardiac disease experiments.

Parameter	Details
Operating System	Windows
Architecture	64 bit
CPU	x86_64
Memory	100 GB
Used Memory	1.52 GB
Environment	Google Colab

**Table 3:** Holistic analysis of Hybrid Random Forest Linear Model.

	Classifier	Accuracy
1	KNN	82.1%
2	LR	82.22%
3	DT	78.22%
4	SVM	82.33%
5	GBM	83.34%
6	RF	80.01%
7	Voting	70.94%
8	Stacking	73.95%
9	RF + KNN (HRFLM)	92.95%
10	RF + SVM (HRFLM)	92.52%
11	RF + LR (HRFLM)	92.59%
12	RF + DT (HRFLM)	84.66%
13	RF + RF (HRFLM)	80.72%
14	RF + KNN (Voting)	72.35%
15	RF + SVM (Voting)	72.86%
16	RF + LR (Voting)	72.28%
17	RF + DT (Voting)	63.63%
18	RF + RF (Voting)	71.72%
19	RF + KNN (Stacking)	68.87%
20	EF + SVM (Stacking)	67.79%
21	RF + LR (Stacking)	73.15%
22	RF + DT (Stacking)	69.19%
23	RF + RF (Stacking)	70.78%

**Table 4:** Holistic analysis of existing literature on cardiovascular disease prediction.

Reference	Outperformed Model	Dataset	Key Findings (%)
[32]	KNN	UCI Heart Disease Dataset	85.18
[33]	MLP	CVD Dataset (70,000 attributes)	87.28
[34]	RF	Self-collected	90
[35]	DT	Heart Disease Dataset	86
[38]	Apache Kafka	Custom Dataset	92
[39]	CART	Aggregated datasets	87
[40]	CNN-LSTM	UCI Heart Disease Dataset	89
[41]	SVM	Cleveland and Statlog	85
[42]	LR	Cleveland Heart Disease	88.52
[43]	CNN-LSTM	CVD Dataset (70,000 attributes)	74.15
Our	HeartEnsembleNet	CVD Dataset (70,000 attributes)	92.95

## Data Availability

The datasets used in this study are publicly available online. Details on access and sources can be provided upon request.

## References

[1] Kazmi N., Gaunt T.R. (2016). Diagnosis of coronary heart diseases using gene expression profiling; stable coronary artery disease, cardiac ischemia with and without myocardial necrosis. PLoS ONE.

[2] Kapila R., Saleti S. (2025). Federated learning-based disease prediction: A fusion approach with feature selection and extraction. Biomed. Signal Process. Control.

[3] Alghamdi F.A., Almanaseer H., Jaradat G., Jaradat A., Alsmadi M.K., Jawarneh S., Almurayh A.S., Alqurni J., Alfagham H. (2024). Multilayer perceptron neural network with arithmetic optimization algorithm-based feature selection for cardiovascular disease prediction. Mach. Learn. Knowl. Extr..

[4] Perreux Y., Chaix M., Kamp A., Mongeon F.P., Pham M., Boussel L., Henaine R., Dore A., Mondésert B., Di-Filippo S. (2020). Abnormal coronary anatomy in patients with transposition of the great arteries and atrial switch: A predictor of serious cardiac adverse events?. Congenit. Heart Dis..

[5] Hong J.S., Lin C.J., Lin Y.H., Lee C.C., Yang H.C., Meng L.H., Lin T.M., Hu Y.S., Guo W.Y., Chu W.F. (2020). Machine learning application with quantitative digital subtraction angiography for detection of hemorrhagic brain arteriovenous malformations. IEEE Access.

[6] Su J.J., Yu D.S.F. (2021). Effects of a nurse-led eHealth cardiac rehabilitation programme on health outcomes of patients with coronary heart disease: A randomised controlled trial. Int. J. Nurs. Stud..

[7] Rani P., Kumar R., Ahmed N.M.S., Jain A. (2021). A decision support system for heart disease prediction based upon machine learning. J. Reliab. Intell. Environ..

[8] Chowdhury M.N.R., Ahmed E., Siddik M.A.D., Zaman A.U. Heart disease prognosis using machine learning classification techniques. Proceedings of the 2021 6th International Conference for Convergence in Technology (I2CT).

[9] Goud P.S., Sastry P.N., Sekhar P.C. (2024). A novel intelligent deep optimized framework for heart disease prediction and classification using ECG signals. Multimed. Tools Appl..

[10] Almazroi A.A., Aldhahri E.A., Bashir S., Ashfaq S. (2023). A clinical decision support system for heart disease prediction using deep learning. IEEE Access.

[11] Nandakumar P., Subhashini R. (2024). Heart Disease Prediction Using Convolutional Neural Network with Elephant Herding Optimization. Comput. Syst. Sci. Eng..

[12] Mandava M., Vinta S.R. (2024). MDensNet201-IDRSRNet: Efficient cardiovascular disease prediction system using hybrid deep learning. Biomed. Signal Process. Control.

[13] Ogunpola A., Saeed F., Basurra S., Albarrak A.M., Qasem S.N. (2024). Machine learning-based predictive models for detection of cardiovascular diseases. Diagnostics.

[14] Naser M.A., Majeed A.A., Alsabah M., Al-Shaikhli T.R., Kaky K.M. (2024). A Review of Machine Learning’s Role in Cardiovascular Disease Prediction: Recent Advances and Future Challenges. Algorithms.

[15] Virmani D., Ghori M.A.S., Tyagi N., Ambilwade R., Patil P.R., Sharma M. Machine Learning: The Driving Force Behind Intelligent Systems and Predictive Analytics. Proceedings of the 2024 International Conference on Trends in Quantum Computing and Emerging Business Technologies.

[16] Ulianova S. (2023). Cardiovascular Disease Dataset. https://www.kaggle.com/datasets/sulianova/cardiovascular-disease-dataset.

[17] Mienye I.D., Jere N. (2024). Optimized ensemble learning approach with explainable AI for improved heart disease prediction. Information.

[18] Sujitha S., Anita C., Sudharson K., Rose S.R., Sharmila S., Keerthana V. OptiANN-LR: Augmenting Diabetes Prediction Accuracy through Hyper Learning Rate Tuning in Optimized Artificial Neural Networks. Proceedings of the 2024 IEEE International Students’ Conference on Electrical, Electronics and Computer Science (SCEECS).

[19] Abuzneid M.A., Mahmood A. (2018). Enhanced human face recognition using LBPH descriptor, multi-KNN, and back-propagation neural network. IEEE Access.

[20] Abdullah D.M., Abdulazeez A.M. (2021). Machine learning applications based on SVM classification a review. Qubahan Acad. J..

[21] Xu Z., Shen D., Nie T., Kou Y. (2020). A hybrid sampling algorithm combining M-SMOTE and ENN based on Random forest for medical imbalanced data. J. Biomed. Inform..

[22] Siddiqui E.F., Ahmed T., Nayak S.K. (2024). A decision tree approach for enhancing real-time response in exigent healthcare unit using edge computing. Meas. Sens..

[23] Wazery Y.M., Saber E., Houssein E.H., Ali A.A., Amer E. (2021). An efficient slime mould algorithm combined with k-nearest neighbor for medical classification tasks. IEEE Access.

[24] Trang N.T.H., Long K.Q., An P.L., Dang T.N. (2023). Development of an artificial intelligence-based breast cancer detection model by combining mammograms and medical health records. Diagnostics.

[25] MurtiRawat R., Panchal S., Singh V.K., Panchal Y. Breast Cancer detection using K-nearest neighbors, logistic regression and ensemble learning. Proceedings of the 2020 International Conference on Electronics and Sustainable Communication Systems (ICESC).

[26] Gonzalez R., Saha A., Campbell C.J., Nejat P., Lokker C., Norgan A.P. (2024). Seeing the random forest through the decision trees. Supporting learning health systems from histopathology with machine learning models: Challenges and opportunities. J. Pathol. Inform..

[27] Sabahno H., Amiri A. (2023). New statistical and machine learning based control charts with variable parameters for monitoring generalized linear model profiles. Comput. Ind. Eng..

[28] Mahajan P., Uddin S., Hajati F., Moni M.A. (2023). Ensemble learning for disease prediction: A review. Healthcare.

[29] Zaidi S.A.J., Tariq S., Belhaouari S.B. (2021). Future Prediction of COVID-19 Vaccine Trends Using a Voting Classifier. Data.

[30] Aljaaf A.J., Al-Jumeily D., Hussain A.J., Dawson T., Fergus P., Al-Jumaily M. Predicting the likelihood of heart failure with a multi level risk assessment using decision tree. Proceedings of the 2015 Third International Conference on Technological Advances in Electrical, Electronics and Computer Engineering (TAEECE), IEEE.

[31] El-Hasnony I.M., Elzeki O.M., Alshehri A., Salem H. (2022). Multi-label active learning-based machine learning model for heart disease prediction. Sensors.

[32] Yadav A.L., Soni K., Khare S. Heart diseases prediction using machine learning. Proceedings of the 2023 14th International Conference on Computing Communication and Networking Technologies (ICCCNT).

[33] Bhatt C.M., Patel P., Ghetia T., Mazzeo P.L. (2023). Effective heart disease prediction using machine learning techniques. Algorithms.

[34] Hossain M.I., Maruf M.H., Khan M.A.R., Prity F.S., Fatema S., Ejaz M.S., Khan M.A.S. (2023). Heart disease prediction using distinct artificial intelligence techniques: Performance analysis and comparison. Iran J. Comput. Sci..

[35] Ishaq A., Sadiq S., Umer M., Ullah S., Mirjalili S., Rupapara V., Nappi M. (2021). Improving the prediction of heart failure patients’ survival using SMOTE and effective data mining techniques. IEEE Access.

[36] Nomali M., Khalili D., Yaseri M., Mansournia M.A., Ayati A., Navid H., Nedjat S. (2023). Validity of the models predicting 10-year risk of cardiovascular diseases in Asia: A systematic review and prediction model meta-analysis. PLoS ONE.

[37] Chicco D., Jurman G. (2020). Machine learning can predict survival of patients with heart failure from serum creatinine and ejection fraction alone. BMC Med Inform. Decis. Mak..

[38] Ozcan M., Peker S. (2023). A classification and regression tree algorithm for heart disease modeling and prediction. Healthc. Anal..

[39] Sudha V., Kumar D. (2023). Hybrid CNN and LSTM network for heart disease prediction. SN Comput. Sci..

[40] Ogundepo E.A., Yahya W.B. (2023). Performance analysis of supervised classification models on heart disease prediction. Innov. Syst. Softw. Eng..

[41] Manikandan G., Pragadeesh B., Manojkumar V., Karthikeyan A., Manikandan R., Gandomi A.H. (2024). Classification models combined with Boruta feature selection for heart disease prediction. Inform. Med. Unlocked.

[42] Mohapatra S., Maneesha S., Mohanty S., Patra P.K., Bhoi S.K., Sahoo K.S., Gandomi A.H. (2023). A stacking classifiers model for detecting heart irregularities and predicting Cardiovascular Disease. Healthc. Anal..

[43] Hossain M.M., Ali M.S., Ahmed M.M., Rakib M.R.H., Kona M.A., Afrin S., Islam M.K., Ahsan M.M., Raj S.M.R.H., Rahman M.H. (2023). Cardiovascular disease identification using a hybrid CNN-LSTM model with explainable AI. Inform. Med. Unlocked.

